# Supplementary material for: Estimating the Direct Effect between Dietary Macronutrients and Cardiometabolic Disease, Accounting for Mediation by Adiposity and Physical Activity
Source: Nutrients. 2022 Mar 13;14(6):1218. doi: 10.3390/nu14061218 (PMC8949537; doi:10.3390/nu14061218)
Supplement: Supplementary file 1 [file nutrients-14-01218-s001.zip › Supplementary_figures.pdf]

Supporting Material

Estimating the direct effect between dietary macronutrients and cardiometabolic disease, accounting for mediation by adiposity and physical activity.

Hugo Pomares-Millan et al.

Abbreviations used:

2h glucose
two-hour glucose
CVD
cardiovascular disease
DE
Direct effect
FG
fasting glucose
GWAS
Genome-wide association analysis
ID
Indirect effect
ICD
International Classification of Diseases
PC
Principal component
TC
Total cholesterol
TE
Total effect
TEI
total energy intake
TG
triglycerides
T2D
type 2 diabetes
VHU
Västerbotten Health Survey

SUPPLEMENTARY FIGURES

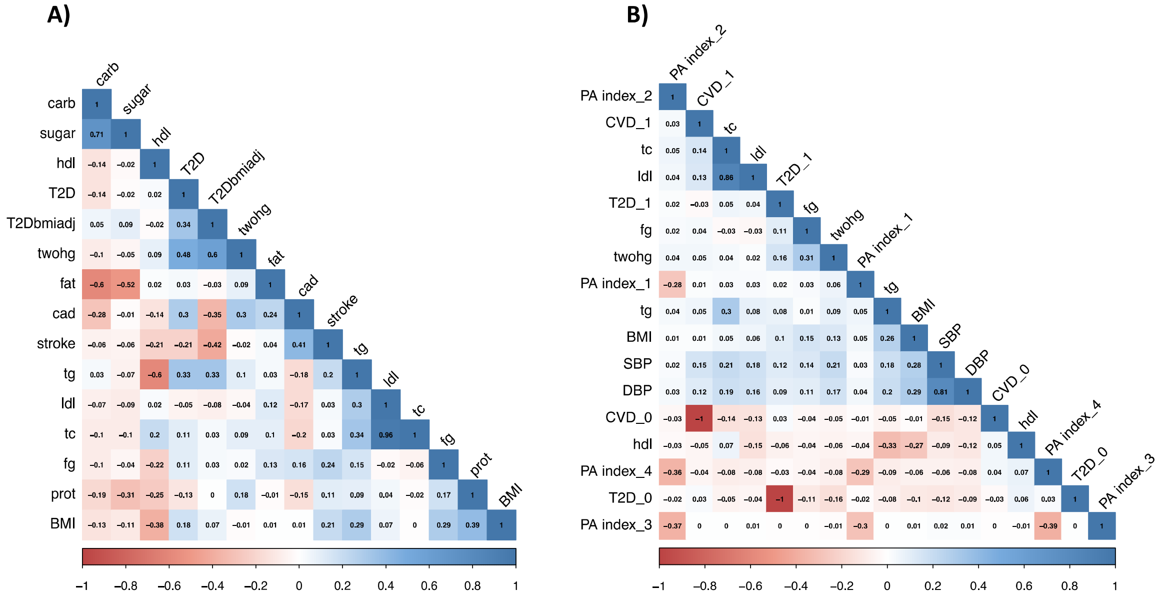

Figure S1. A) Genotypic and B) phenotypic correlations. Genotypic correlations were computed using Linkage Disequilibrium (LD) score regression [1] and phenotypic are Pearson's pairwise-correlations. The blue colour represents positive correlations and red represents negative correlations. Carb: carbohydrate intake (E%); Prot: protein intake (E%); PA index (categorical): Physical activity index; T2D1: Type 2 diabetes: Yes; T2D0: Type 2 diabetes: No; CAD: coronary artery disease; CVD1: Cardiovascular disease: Yes; CVD0: Cardiovascular disease: No; HDL: high-density lipoprotein; LDL: low-density lipoprotein; TG: triglycerides; TC: total cholesterol; SBP: systolic blood pressure; DBP: diastolic blood pressure; FG: fasting glucose; 2h glucose: two-hour glucose; BMI: body mass index.

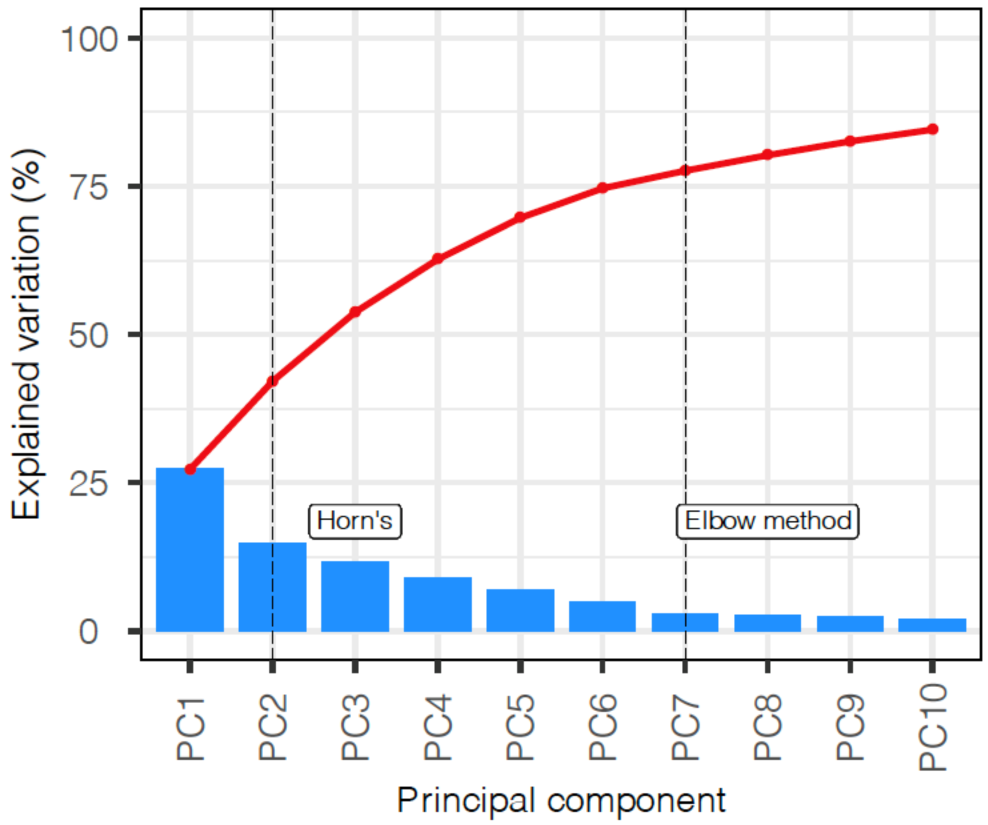

Figure S2. Screeplot of percentage of variance explained by the first 10 PCs with Horn's and elbow optimal number of PCs.

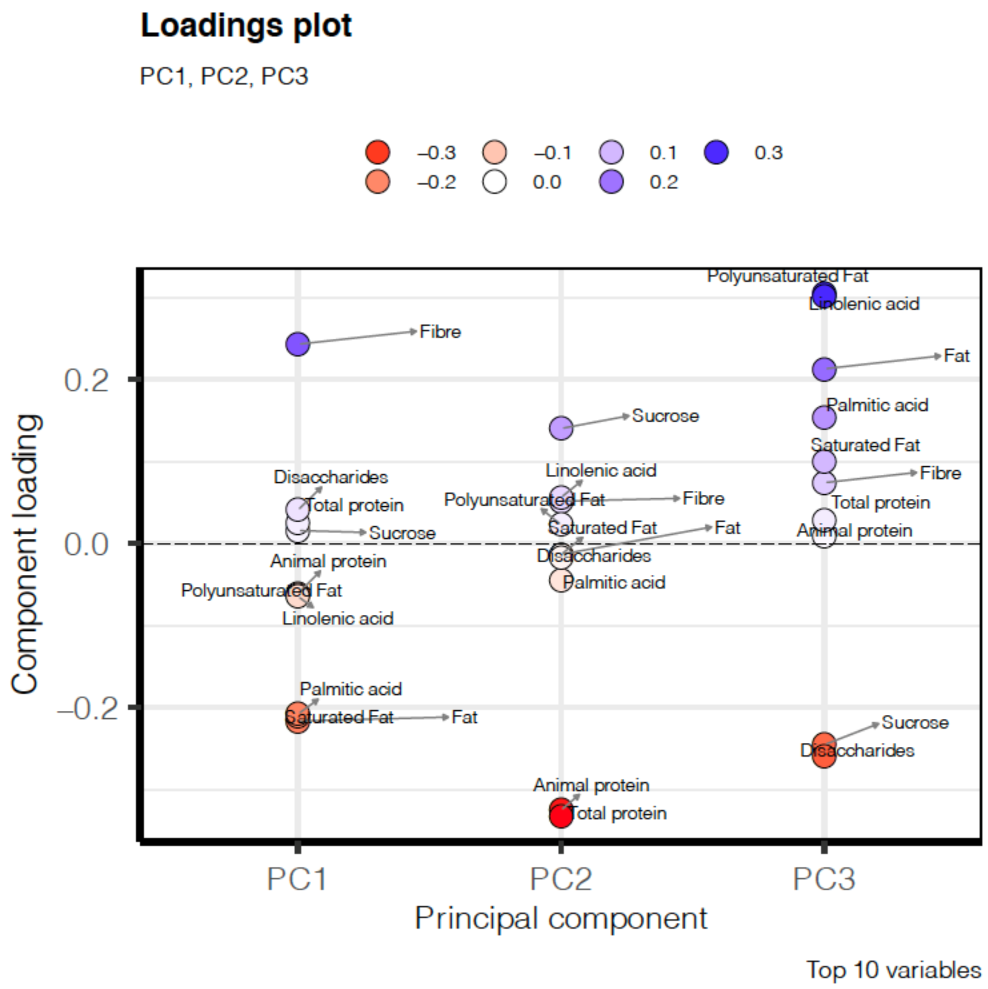

Figure S3. The top 10 variables contributing the most to the top 3 PC-DPs.

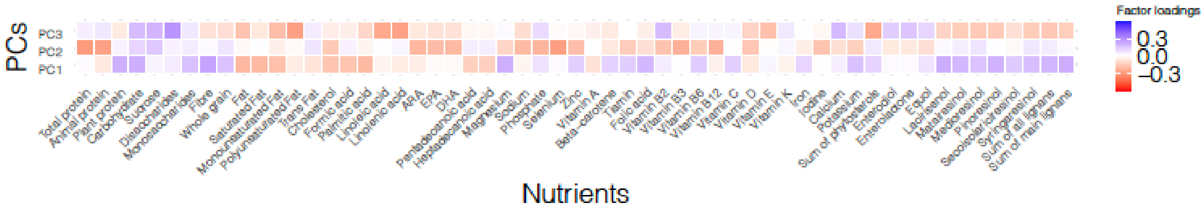

Figure S4. Nutrient variables correlation and top 3 PC-DPs.

Two sample mendelian randomization and Bayesian colocalization

As sensitivity analyses, we conducted MR-Egger and weighted median regressions to assess if yielded consistent results, moreover, we performed a leave-one-out analysis (Figure S5). When

the SNP rs4420638 was removed, which was significantly associated between carbohydrate intake (E%) and T2DadjBMI ($p < 9.19E-04$), the significance remained, β_{IVW} -0.62 (95% CI: -1.14, -0.01; p= 0.02), $\beta_{MR\text{-}Egger}$ -1.52 (95% CI: -3.02, -0.01; p= 0.049) and $P_{MR\text{-}PRESSO}$= 0.512; In addition, we tested the opposite direction (reverse causation) of the association, i.e., T2DadjBMI as exposure and carbohydrate intake (E%) as outcome, with no significant association (Table S25).

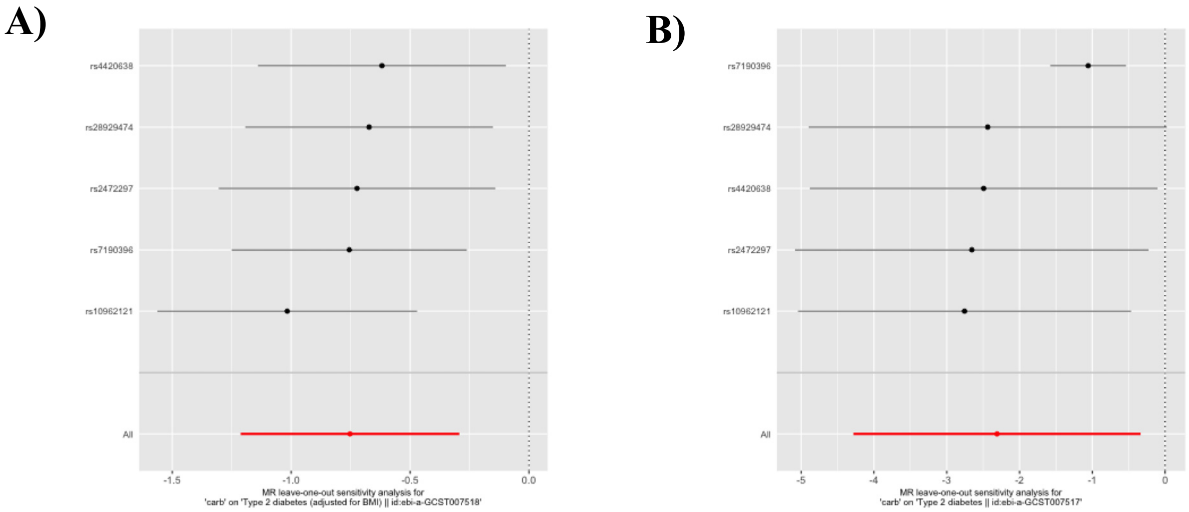

Figure S5. Leave-one-out sensitivity analysis for A) T2D adjusted for BMI instruments and B) T2D instruments.

Bayesian colocalization is a deterministic approach that assumes that in each region, there is at most one causal SNP for either trait (i.e. single causal variant assumption) or a variant in very strong LD with the true causal variant. We used the GWAS-significant instruments for T2D [2] to align with macronutrient intake traits (Table S30). Whether dietary traits are genetically correlated or not [3], it is conceivable each one may have a unique or a shared genetic architecture.

To further explore the genetically predicted macronutrient intake and T2D, we conducted pairwise and multi-trait colocalization of shared causal variants between T2D, adjusted and unadjusted for BMI, carbohydrate, sugar, protein, and fat intake. Considering SNPs are required

to be associated with T2D irrespective of the causal configuration, we used the 54 and 58 GWAS-significant SNPs as index to align and colocalize (Table S30). The T2DadjBMI and the macronutrient intake colocalized (posterior probability >0.9) in the region 10, position: 112998590 at *rs7903146* near the known *TCF7L2* locus [4,5] , a variant reported to have a T-allele associated with T2D incidence [6] and interaction with carbohydrate intake in the forms of fibre [7], high-fat low-carbohydrate diet [8], and whole-grain intake [9]. The T2D and macronutrient intake colocalized in the region 16, position: 53769662 with candidate *rs1558902* (posterior probability >0.9) associated with *FTO* gene [10]. When we tested pairwise colocalization between T2D or T2DadjBMI and macronutrient intake using 'HyPrColoc' and 'coloc' packages, *rs7903146* remained as single candidate variant when colocalized with carbohydrates, yet no posterior probability surpassed the threshold (>0.7). As recommended by the authors of the 'HyPrColoc' package, we tested the prior probability (p_1) that a single SNP is associated with a single trait 1 in 1000 [11], and p_1 = 1e-10, 1e-15, 1e-20, and 1e-25. Thus, with the conditional variant-specific prior of 1e-10, we performed colocalization with additional prior probabilities (p_c) for each additional trait with p_c = 0.05, 0.02, 0.01, and 0.005 with no material changes [12].

References:

1. Bulik-Sullivan, B.K.; Loh, P.-R.; Finucane, H.K.; Ripke, S.; Yang, J.; Patterson, N.; Daly, M.J.; Price, A.L.; Neale, B.M. LD Score regression distinguishes confounding from polygenicity in genome-wide association studies. *Nature genetics* **2015**, *47*, 291-295.
2. Mahajan, A.; Wessel, J.; Willems, S.M.; Zhao, W.; Robertson, N.R.; Chu, A.Y.; Gan, W.; Kitajima, H.; Taliun, D.; Rayner, N.W., et al. Refining the accuracy of validated target identification through coding variant fine-mapping in type 2 diabetes. *Nat Genet* **2018**, *50*, 559-571, doi:10.1038/s41588-018-0084-1.
3. Merino, J.; Dashti, H.S.; Li, S.X.; Sarnowski, C.; Justice, A.E.; Graff, M.; Papoutsakis, C.; Smith, C.E.; Dedoussis, G.V.; Lemaitre, R.N. Genome-wide meta-analysis of macronutrient intake of 91,114 European ancestry participants from the cohorts for heart and aging research in genomic epidemiology consortium. *Molecular psychiatry* **2019**, *24*, 1920-1932.
4. Grant, S.F.; Thorleifsson, G.; Reynisdottir, I.; Benediktsson, R.; Manolescu, A.; Sainz, J.; Helgason, A.; Stefansson, H.; Emilsson, V.; Helgadottir, A., et al. Variant of

transcription factor 7-like 2 (TCF7L2) gene confers risk of type 2 diabetes. *Nat Genet* **2006**, *38*, 320-323, doi:10.1038/ng1732.
5. Zeggini, E.; Scott, L.J.; Saxena, R.; Voight, B.F.; Marchini, J.L.; Hu, T.; de Bakker, P.I.; Abecasis, G.R.; Almgren, P.; Andersen, G., et al. Meta-analysis of genome-wide association data and large-scale replication identifies additional susceptibility loci for type 2 diabetes. *Nat Genet* **2008**, *40*, 638-645, doi:10.1038/ng.120.
6. Florez, J.C.; Jablonski, K.A.; Bayley, N.; Pollin, T.I.; de Bakker, P.I.; Shuldiner, A.R.; Knowler, W.C.; Nathan, D.M.; Altshuler, D. TCF7L2 polymorphisms and progression to diabetes in the Diabetes Prevention Program. *N Engl J Med* **2006**, *355*, 241-250, doi:10.1056/NEJMoa062418.
7. Hindy, G.; Sonestedt, E.; Ericson, U.; Jing, X.-J.; Zhou, Y.; Hansson, O.; Renström, E.; Wirfält, E.; Orho-Melander, M. Role of TCF7L2 risk variant and dietary fibre intake on incident type 2 diabetes. *Diabetologia* **2012**, *55*, 2646-2654.
8. Grau, K.; Cauchi, S.; Holst, C.; Astrup, A.; Martinez, J.A.; Saris, W.H.; Blaak, E.E.; Oppert, J.-M.; Arner, P.; Rössner, S. TCF7L2 rs7903146–macronutrient interaction in obese individuals' responses to a 10-wk randomized hypoenergetic diet. *The American journal of clinical nutrition* **2010**, *91*, 472-479.
9. Fisher, E.; Boeing, H.; Fritsche, A.; Doering, F.; Joost, H.-G.; Schulze, M.B. Whole-grain consumption and transcription factor-7-like 2 (TCF7L2) rs7903146: gene–diet interaction in modulating type 2 diabetes risk. *British Journal of Nutrition* **2008**, *101*, 478-481.
10. Garver, W.S.; Newman, S.B.; Gonzales-Pacheco, D.M.; Castillo, J.J.; Jelinek, D.; Heidenreich, R.A.; Orlando, R.A. The genetics of childhood obesity and interaction with dietary macronutrients. *Genes & nutrition* **2013**, *8*, 271-287.
11. Giambartolomei, C.; Vukcevic, D.; Schadt, E.E.; Franke, L.; Hingorani, A.D.; Wallace, C.; Plagnol, V. Bayesian test for colocalisation between pairs of genetic association studies using summary statistics. *PLoS genetics* **2014**, *10*, e1004383.
12. Wallace, C. Eliciting priors and relaxing the single causal variant assumption in colocalisation analyses. *PLoS genetics* **2020**, *16*, e1008720.
